# Repetitive Breech Presentations at Term

**DOI:** 10.1155/2013/628572

**Published:** 2013-07-30

**Authors:** Pavol Zubor, Imrich Zigo, Jana Sivakova, Petra Moricova, Ivana Kapustova, Stefan Krivus, Jan Danko

**Affiliations:** Department of Obstetrics and Gynecology, Jessenius Faculty of Medicine, Comenius University, Kollarova 2, 03601 Martin, Slovakia

## Abstract

The authors present a case of 38-year-old laboring woman with four-time repetitive breech presentation of the fetus at term. This rare condition affects the mode of delivery and represents serious obstetrical problem as it is associated with increased perinatal morbidity or mortality. The authors give details on risk factors for breech presentation, its diagnosis, and the discussion points on possible causes leading to repetitive breeches in laboring women.

## 1. Introduction

The breech presentation is defined as the initial entrance of the gluteal region of the fetus into the maternal pelvis and is the most common abnormal fetal presentation. Breech delivery is a challenge in obstetric management and is associated with increased perinatal morbidity and mortality [1–3]. The prevalence of breeches ranges from 3% to 4% at term [4]. The predisposing factors for breech presentation are prematurity, multiple gestation, multiparity, fetal hydrocephalus, oligohydramnios, polar placentation, placenta previa, gestational diabetes, history of breech delivery, short umbilical cord, low birth weight, uterine anomalies, congenital anomaly, previous cesarean delivery, and pelvic tumors. As these factors explain only 15% of the variance of breech presentation [5–7], the etiology of breech delivery (causes of failure of spontaneous cephalic version) is not clear. Moreover, when fetal malposition in the form of breech presentation is repetitively presented in the same women. Thus, further research into the mechanism of breech presentation is needed.

 The current study describes unusual case of repetitive (4 times) recurrence of breech delivery in the same women with hypothesis describing possible etiology of such rare case.

## 2. Case Report

**Figure 1:**
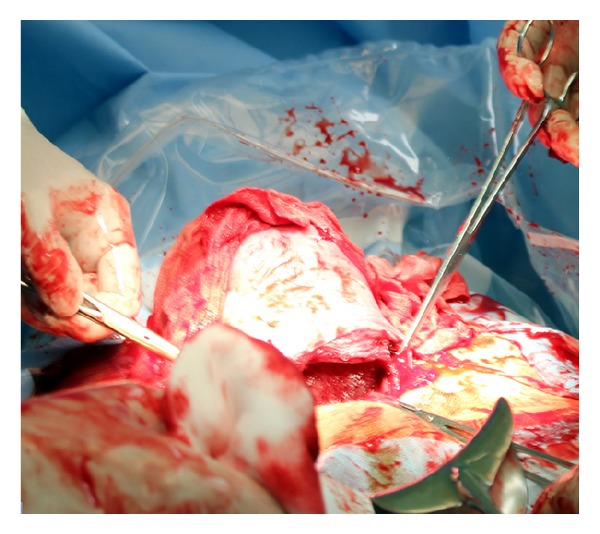
The intraoperative findings on the uterus.

 A 38-years-old quadrugravida 168 cm tall Caucasian woman with singleton pregnancy at term (38 + 5 g.w.) was admitted to the delivery room due to regular uterine contractions every 5 minutes and sharp pain perception in scar after previous cesarean section. Gynecology examination revealed breech (Frank) presentation and vaginal findings showed 1 cm long cervix, dilated to 3 cm without signs of bleeding. A fetal predelivery ultrasonography was done with term corresponding biometry and EFW 3420 grams. The fetal monitor consisted of a baseline 140 bpm, accelerations, and good variability. Increased pain perception in lower uterine segment below the scar was the reason for pregnancy termination by uncomplicated cesarean section (Joel-Cohen incision with Misgav Ladach method) 3.5 hrs after admission. The intraoperative finding on the uterus was negative, without myoma, septum, or adhesions (Figure 1). Her past history was uneventful except the obstetrical history of repetitive breech presentation of all previous deliveries (2 times spontaneously, once by low transverse cesarean section, Table 1). A 3500 g weighted male neonate with 9, 10, 10 Apgar score was delivered and discharged home with mother on 5th day after uncomplicated postpartum recovery.

## 3. Discussion

The breech delivery may represent the obstetrical problem that necessitates increased patience because of it possible complications. Thus many pregnancies with fetuses in breech lie are terminated for prevention by cesarean section. On the other side there exist obstetricians that can deliver breeches without fetal injury. However, despite their wide clinical practice, few of them faced the situation as described by our case: four-time repetitive breech presentation in one laboring woman giving the probability of such event at 0.045. In this section we will try to explain some possible reasons of this rare obstetric situation.

There are several maternal and fetal factors that predispose to a breech presentation, for example, uterine anomalies, myomas, pelvic tumors, fetal anomalies, changes of amniotic fluid, placental localization, or length of umbilical cord [8] that solely or in combination provoke fetal malpresentation. Moreover, some studies have found a relationship between placenta previa and polar fundal localization of the placenta and the breech presentation [9, 10]. For example, cornu-fundal localization of the placenta occurred in 70% of breech presentations, but only in about 5% of cephalic presentations. In our case we have revealed 3-time fundal and one anterior wall placenta localizations on antepartum ultrasound what confirms the above-mentioned risk factors for breech presentations. 

Adinma's study, in which 1000 cases were observed, found that breech fetuses had shorter umbilical cords than those of cephalic fetuses and that the average umbilical cord length is about 51 cm (range, 15–130) [11]. The lengths of umbilical cords in our case were as follows for subsequent pregnancies: 34 cm, 53 cm, 42 cm, and 57 cm, respectively. The relative short cords in pregnancies may explain the repetitive breeches in our case. 

Specificity for higher breech presentation is body mass index (BMI) and maternal weight gain during pregnancy. It was revealed that higher BMI at term and increased body weight gain might be related to persistent breech presentation [12]. The last pregnancy of our case was associated with 13 kg body weight gain and 34 kg/m^2^ BMI score. Another study revealed significantly higher incidence of breech deliveries in fetuses with increased placenta weight; however the reason was not revealed [13]. The weights of placentas in our case were in the range 430–560 g what represent the normal findings. Thus, did not confirm the above presented reasons associated with an increased risk for breech presentation.

Rayl et al. [14] highlighted smoking as a risk factor for breech presentation. In the current study we found no smoking history in any of the presented pregnancies. Similarly, Vendittelli et al. [15] reported that women with previous cesarean deliveries were at twice the risk of breech presentation at term than women with previous vaginal deliveries, which was in agreement with our data. Interestingly, Kuppens et al. [16] have provided data that women with higher TSH levels (>2.5 mIU/L) in pregnancy may exhibit higher rate of breech deliveries. This cannot be confirmed by our case as we did not make a hormonal profile in all of the pregnancies. 

Nordtveit et al. [17] showed that breech delivery may exhibit partly inherited patterns as both men and women delivered in breech presentation at term contribute to increased risk of breech delivery in their offspring. Thus, this supports the theory that some genes on the specific environmental background passed on from the father or the mother seem to be closely related to breech delivery. Furthermore, the congenital pattern of breech delivery is supported by the findings from race-targeted studies, which have proved that white women have 69% higher risk for breech delivery than black women [18].

Finally, the congenital disorders in cerebellum cannot be omitted in the fetuses when describing the reason for breech delivery [19]. However, this theory has to be proved in the future. In general, there exists approximately 15% risk of repetitive breech delivery after the previous one, and this risk is higher for cesarean sections [20]. The relative risk of breech recurrence in a second pregnancy is 3.2 and in a third consecutive breech pregnancy 13.9 [21]. Any other breeches are extremely rare. Thus our case of four forthcoming breeches are worthy for presentation and for closer investigation of the causes. 

In summary, we conclude that shorter umbilical cord, higher placental weight, increased maternal term BMI, cornu-fundal placenta localization, smoking, thyroid hormone disbalance, race, and genetics may be causative factors for repetitive breech presentations as described by our case and that it is the persistence of maternal risk factors rather than first pregnancy fetal or infant factors that play a role in repeating breech presentations at term. Thus, the consistently elevated recurrence rates highlight the need for women with a history of breech delivery to be closely monitored in the later stages of pregnancy (e.g., attempt for external cephalic version or prevention of cord prolapse after PROM).

## Figures and Tables

**Table 1:** The clinical data of all deliveries.

Parameter/pregnancy	1st pregnancy	2nd pregnancy	3rd pregnancy	4th pregnancy
Gest. week at delivery	40	38	41	39
Mode of delivery	Vaginal	Vaginal	CS	CS
Birth weight (gram)	3260	3370	3940	3500
Birth length (cm)	51	50	54	52
Gender	Female	Female	Female	Male
Apgar score	8/9/9	8/8/9	9/9/9	9/10/10
Placenta localization	Fundal	Anterior	Fundal	Fundal
Placenta weight (gram)	520	430	560	490
Umbilical cord length (cm)	34	53	42	57
BMI at delivery (kg/m^2^)	30.4	36.5	34.3	33.9
Body weight gain (kg)	15	19	14	13
Smoking history	No	No	No	No
Congenital disorders	No	No	No	No
PROM	No	Yes	No	No
Serology (TORCH + TPPA)	Negative	Negative	Negative	Negative
3rd trimester maternal Hb	119	105	128	115

## Abbreviations

EFW: Estimated fetal weight
Bpm: Beats per minute
PROM: Premature rupture of membranes
TORCH: Toxoplasmosis, other (syphilis, varicella-zoster, and parvovirus B19), *Rubella*, *Cytomegalovirus* (CMV), and *Herpes* infections
CS: Caesarean section
TPPA: *Treponema pallidum* particle agglutination assay
Hb: Hemoglobin
BMI: Body mass index.
